# Case report and literature review: novel TXNRD2 compound heterozygous variants in familial glucocorticoid deficiency type 5

**DOI:** 10.3389/fped.2025.1585582

**Published:** 2025-07-14

**Authors:** Xiaoyan Wang, Xiuli Chen, Ting Chen, Rongrong Xie, Qi Lin Chen, Haiying Wu, Fengyun Wang

**Affiliations:** Department of Endocrinology, Genetics and Metabolism, Children's Hospital of Soochow University, Suzhou, China

**Keywords:** FGD5, TXNRD2, exome sequencing, qPCR, glucocorticoid deficiency

## Abstract

Familial glucocorticoid deficiency (FGD) is a rare autosomal recessive disorder characterized by isolated glucocorticoid deficiency. Mutations of *MC2R*, *MRAP*, *STAR*, *NNT*, and *TXNRD2* have been implicated in FGD pathogenesis. To date, only four families with *TXNRD2-*associated familial Glucocorticoid Deficiency Type 5 (FGD5) have been reported worldwide. We report a patient with clinical features consistent with FGD5, increasing the total number of reported cases. Including this case, 11 probands across five independent kindreds have now been identified globally. Functional studies demonstrated that the novel compound heterozygous variants (c.1391A > G; p.H464R and c.1141C > T; p.R381W) reduce *TXNRD2* protein levels in a heterologous expression system. This case expands the genetic spectrum of FGD5 and suggests a potential association between *TXNRD2* variants and electrocardiographic abnormalities. Our findings underscore the importance of *TXNRD2* in adrenal redox homeostasis and provide new insights for FGD5 diagnosis.

## Background

Familial glucocorticoid deficiency (FGD), a rare hereditary endocrine disorder, manifests as selective adrenal unresponsiveness to adrenocorticotropic hormone (ACTH), resulting in isolated cortisol deficiency with preserved aldosterone synthesis. This condition typically presents in early childhood with life-threatening complications including hypoglycemic seizures, growth retardation, and characteristic mucocutaneous hyperpigmentation secondary to excessive ACTH-driven melanocyte stimulation. The characterized comprises undetectable serum cortisol, markedly elevated ACTH levels, and normal renin-aldosterone axis activity (1). FGD is characterized by undetectable serum cortisol, markedly elevated ACTH levels, and normal renin-aldosterone axis activity. It is primarily recognized by glucocorticoid deficiency alone, with other less common clinical manifestations including salt loss and hypoaldosteronism, primary hypothyroidism, hypogonadism, and growth defects (2).

Molecular studies have delineated nine causative genes associated with FGD, which can be functionally classified into three categories: ACTH Receptor Complex Defects: Pathogenic variants in *MC2R* (encoding melanocortin 2 receptor) and its accessory protein *MRAP* account for approximately 45% of cases. Mitochondrial Redox Dysregulation: Mutations affecting antioxidant defense systems, including *NNT*, *TXNRD2*, and *GPX1*, contribute to adrenal oxidative stress and steroidogenic failure. Steroid Biosynthesis Impairment: Rare variants in STAR and CYP11A1 disrupt cholesterol transport and initial steroidogenesis steps. The *TXNRD2* gene encodes a mitochondrial selenoprotein essential for maintaining thioredoxin-mediated redox homeostasis. *TXNRD2* catalyzes the NADPH-dependent reduction of oxidized thioredoxin, a critical antioxidant system protecting adrenal cells from reactive oxygen species(ROS) during steroidogenesis. Since the inaugural description of *TXNRD2*-linked FGD in 2014, global reports remain scarce, with distinctive clinical features. The distinctive clinical features include: neonatal-onset hypermelanosis involving mucosal surfaces, profound glucocorticoid insufficiency, exaggerated ACTH elevation, and intact zona glomerulosa function.

We report a case of thioredoxin reductase 2 (*TXNRD2*)-linked familial glucocorticoid deficiency-5 (FGD5) that occurred in China, increasing the total number of reported probands with this mutation and providing additional phenotypic information of this rare syndrome. In this case, a Chinese family harboured compound heterozygous variants (c.1391A > G; p.H464R and c.1141C > T; p.R381W). Our findings may expand the genetic and clinical spectrum of FGD5. We subsequently provide a review of pathogenic mutations currently documented in *TXNRD2*, including their specific localization across the gene's structural organization and corresponding protein domains, as well as their associated clinical manifestations.

## Materials and methods

### Ethical compliance and clinical examination

All procedures were approved by the Institutional Ethics Board of Children's Hospital of Soochow University(2021CS003), and informed consent was obtained from the patient's parents. Routine blood tests, serum electrolytes, serum biochemistry, thyroid function, plasma cortisol, ACTH, aldosterone, angiotensin II, renin, 24-h urine free cortisol, cellular/humoral immunity, magnetic resonance imaging (MRI) of the brain, adrenal ultrasound, testosterone, and bone age were assessed. All clinical information was collected and reviewed, and all clinical information was collected and investigated. All the procedures were performed in accordance with the Declaration of Helsinki (2013 revision).

### Whole exome sequencing

Blood samples were collected from the patient and his family. Genomic DNA (gDNA) was extracted from the whole blood of the affected children and their parents. DNA samples from the case was fragmented using a Scientz08-III automated sonicator (NingBo Scientz Biotechnology, Ningbo, China) DNA samples were extracted using a DNA extraction kit (CWE2100 Blood DNA kit V2; Beijing Kangwei Century Biotechnology Co., Ltd., Beijing, China) on a 96-channel automatic nucleic acid extraction machine (Beijing Kangwei Century Biotechnology Co., Ltd.). DNA samples (750 ng) were fragmented into 150–200 bp via ultrasound treatment for 35 min (running for 3 s with 1-second intervals) with 50% ultrasonic intensity at 4˚C. High-throughput sequencing was performed on a HiSeq 2,500 system (Illumina, San Diego, CA, USA), and the data were sequenced using a sequencer (Illumina).

Data reading and bioinformatics analysis were performed following Control Software assessment (The Genome Analysis Toolkit; http://www.roadinstitute.org/gsa/wiki/index.php/Home_Page). The obtained sequences were separately Basic Local Alignment Search Tool–matched with GenBank (http://www.ncbi.nlm. nih.gov/genbank) sequences to confirm the variant sites; suspected variant sites were searched using MITOMAP (www. mitomap.org/MITOMAP). For polymorphism detection, gene variants were detected and verified via Sanger sequencing.

For the validation of the panel, parents were analyzed. The detected pathogenicity of rare variants (minor allele frequency ≤0.01) was evaluated according to the recommendations of the American College of Medical Genetics and Genomics (ACMG) for variant classification and reporting (3). Population data were determined using public genomic databases, including the 1,000 Genomes Project (https://www.internationalgenome.org), the Genome Aggregation Database (gnomAD; http://gnomad.broadinstitute.org/about), and the Database of Single Nucleotide Polymorphisms (dbSNP; https://www.ncbi.nlm.nih.gov/SNP/).

Other criteria considered were the type of variant (e.g., frameshift, nonsense, or essential splice variants), and clinical, functional, and genotype–phenotype data from the literature and disease databases, including the Human Gene Mutation Database Professional (http://www.hgmd.cf.ac.uk/ac/index.php) and PubMed (https://www.ncbi.nlm.nih.gov/pubmed). If such variants had not been previously reported, they were evaluated to predict their possible functional significance using in silico prediction tools including SIFT (https://sift.jcvi.org), PolyPhen2 (http://genetics.bwh.harvard.edu/pph2/), and Mutation Taster (http://www.mutationtaster.org).

### RNA extraction and *TXNRD2* qPCR

Total RNA was extracted from blood using the Cell Total RNA Isolation Kit (RC113-01, Vazyme, Nanjing, China) and subsequently reverse transcribed to cDNA through HiScript II 1st Strand cDNA Synthesis Kit (R212-01, Vazyme, Nanjing, China). Gene expression levels were normalized using glyceraldehyde-3-phosphate dehydrogenase (GAPDH). Sample quality was controlled using a Nanodrop ND-1000 spectrophotometer. Quantitative qPCR data of mRNA products were analysed by the 2-*ΔΔ*CT method. The primers used for qPCR were as follows: TXNRD2 (forward: 5'-TTGAGGTCTATCACGCCC-3'; reverse:5'-CTTGAGTAACTTCGCCTGC-3') and GAPDH (forward: 5'-CTGGGCTACACTGAGCACC-3’; reverse: 5'-AAGTGGTCGTTGAGGGCAATG-3').

### Western blot

Peripheral blood mononuclear cells (PBMCs) were isolated using density gradient centrifugation through whole blood. The proteins were extracted from PBMC using the RIPA lysis and extraction buffer (P0013B, Beyotime, Shanghai, China). The protein levels were quantified using a bicinchoninic acid (BCA) assay kit (P0010; Beyotime, Shanghai, China). The membrane was blocked with 5% skim milk for two hours and then incubated overnight at 4°C with GAPDH Mouse Monoclonal Antibody (1:1,000, AF0006, Beyotime, China) and TXNRD2 Polyclonal Antibody (1:1,000, 16,360-1-AP, Proteintech, USA). After washing, the membrane was incubated for one hour at room temperature with horseradish peroxidase (HRP)-conjugated goat anti-mouse/rabbit IgG (H + L) (1:5,000, A0216/A0208, Beyotime, China) as appropriate. Detection was performed using an enhanced chemiluminescence (ECL) system.

### Protein structural modeling

To predict the structural changes of the identified mutation, Both wild-type and mutant *TXNRD2* pdb files were predicted by AlphaFold developed by the Deepmind team, the data were realized and presented using Chimera software. The thermodynamic stability was predicted using the DynaMut online prediction tool (https://biosig.lab.uq.edu.au/dynamut/).

### Statistical analyses

Data from independent experiments were presented as mean ± SEM. qPCR results were analyzed using a nonparametric test, and *p* < 0.05 were considered statistically significant. The significance between groups was calculated using GraphPad Prism 8 (GraphPad, USA).

## Results

### Case presentation

A 7-year-old Chinese male presented to our institution in May 2024 with acute gastroenteritis. During hospitalization, generalized hyperpigmentation was noted on physical examination. The patient had exhibited progressive skin discolouration of the lips and fingertips over the preceding week and had a history of congenital cutaneous hypermelanosis since birth. The patient had no history of drug exposure, trauma, dysphagia, vomiting, or chronic medical conditions (neurological, cardiac, renal, or hepatic).

Perinatal history: Born full-term via cesarean section for nuchal cord, with no prenatal/natal asphyxia or neonatal distress.

Developmental milestones: Age-appropriate.

**Figure 1:**
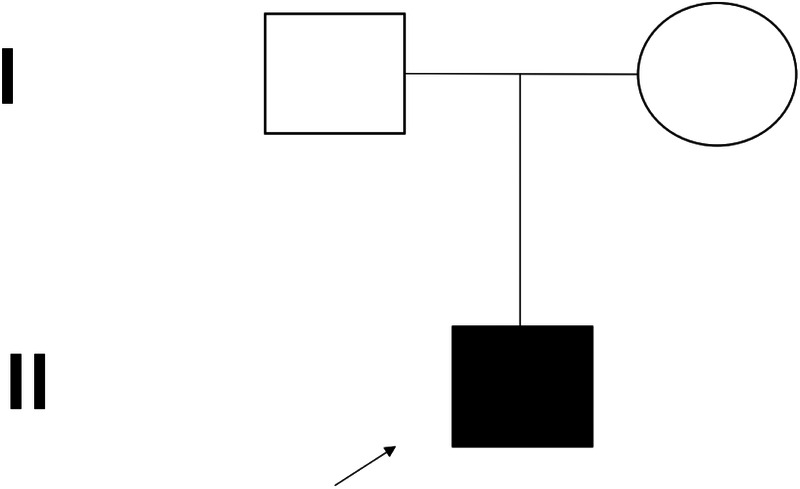
The family pedigrees of the FGD5 (*TXNRD2* c.1391A>G and c.1141C>T).

Family history: No consanguinity or tuberculosis exposure (Figure 1).

Physical Examination: Height 130 cm, weight 30 kg. Heart rate 116 bpm, blood pressure 104/80 mmHg, respiratory rate 26/min. Diffuse hyperpigmentation with accentuation on extensor surfaces (elbows, knees), palmar creases, and buccal mucosa. Normal genitalia, lacrimation, and primitive reflexes. No dysmorphic features or neurologic deficits.

Testosterone: <0.24 nmol/L (reference: 0–1.02 nmol/L) and normal serum electrolytes. The 17ohp level was 262.10 pg/ml (reference: <1,100 pg/ml). Subsequent investigations revealed low serum cortisol (morning: <22 nmol/L, reference 138–690 nmol/L; afternoon: <22 nmol/L, reference 69–345 nmol/L) and very high adrenocorticotropic hormone (ACTH) levels (>278 ng/L, reference <46.37 ng/L) suggesting glucocorticoid deficiency. 24-hour urine free cortisol: 73.40 nmol/24 h (reference: 138.20–1,207.9 nmol/24 h), His serum aldosterone level was normal.

**Figure 2:**
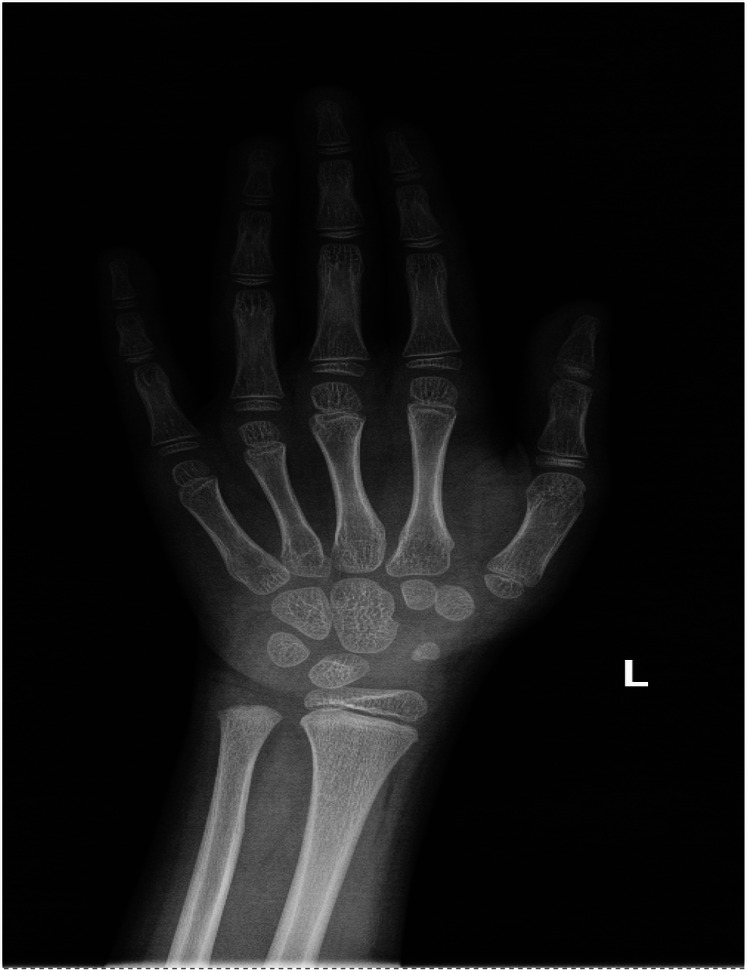
The bone age of the FGD5 (*TXNRD2* c.1391A>G and c.1141C>T).

**Figure 3:**
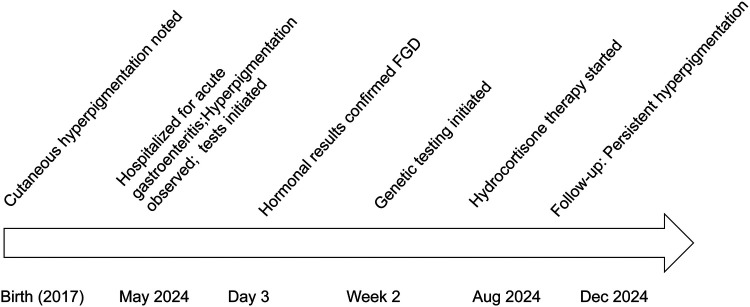
Timeline of the disease course.

**Figure 4:**
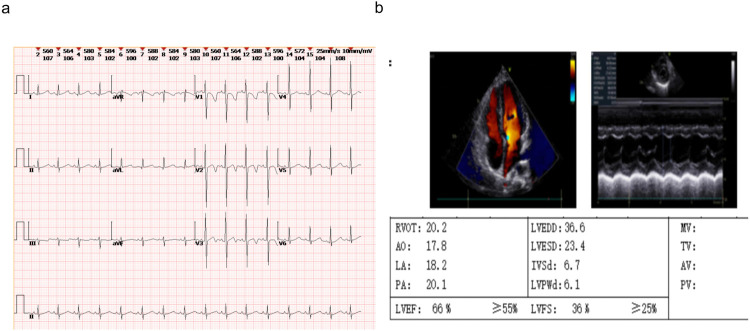
The ECG and echocardiography of the FGD5 (*TXNRD2* c.1391A>G and c.1141C>T). **(a)** ECG, **(b)** echocardiography.

Other tests: Normal complete blood count, renal/liver function, renin-angiotensin system, cellular/humoral immunity, and serum electrolytes. Bone age was about 6 years old (Figure 2). Timeline of the disease course (Figure 3). Electrocardiographic Findings: Initial 12-lead electrocardiogram revealed a prolonged corrected QT interval with discernible U waves in precordial leads (Figure 4). 24-hour Holter Monitoring: Predominant sinus rhythm with marked diurnal heart rate variability (range: 68–142 bpm), intermittent cardiac arrhythmias including: Sporadic premature atrial contractions (70 events/24 hr), Paroxysmal atrial tachycardia (3 episodes, maximum duration 8 beats), Dynamic QT interval prolongation with nocturnal accentuation (maximum QTc 520 ms between 01:00 and 03:00). Magnetic resonance imaging of his brain and adrenal ultrasound were normal. Transthoracic echocardiography (TTE) revealed preserved biventricular systolic function (LVEF 66%) with no evidence of valvular abnormalities, chamber dilation, or regional wall motion abnormalities. Contrast-enhanced computed tomography angiography (CTA) of the thoracoabdominal vasculature demonstrated: normal aortic arch branching pattern, no aneurysmal dilation.

### Genetic testing

**Figure 5:**
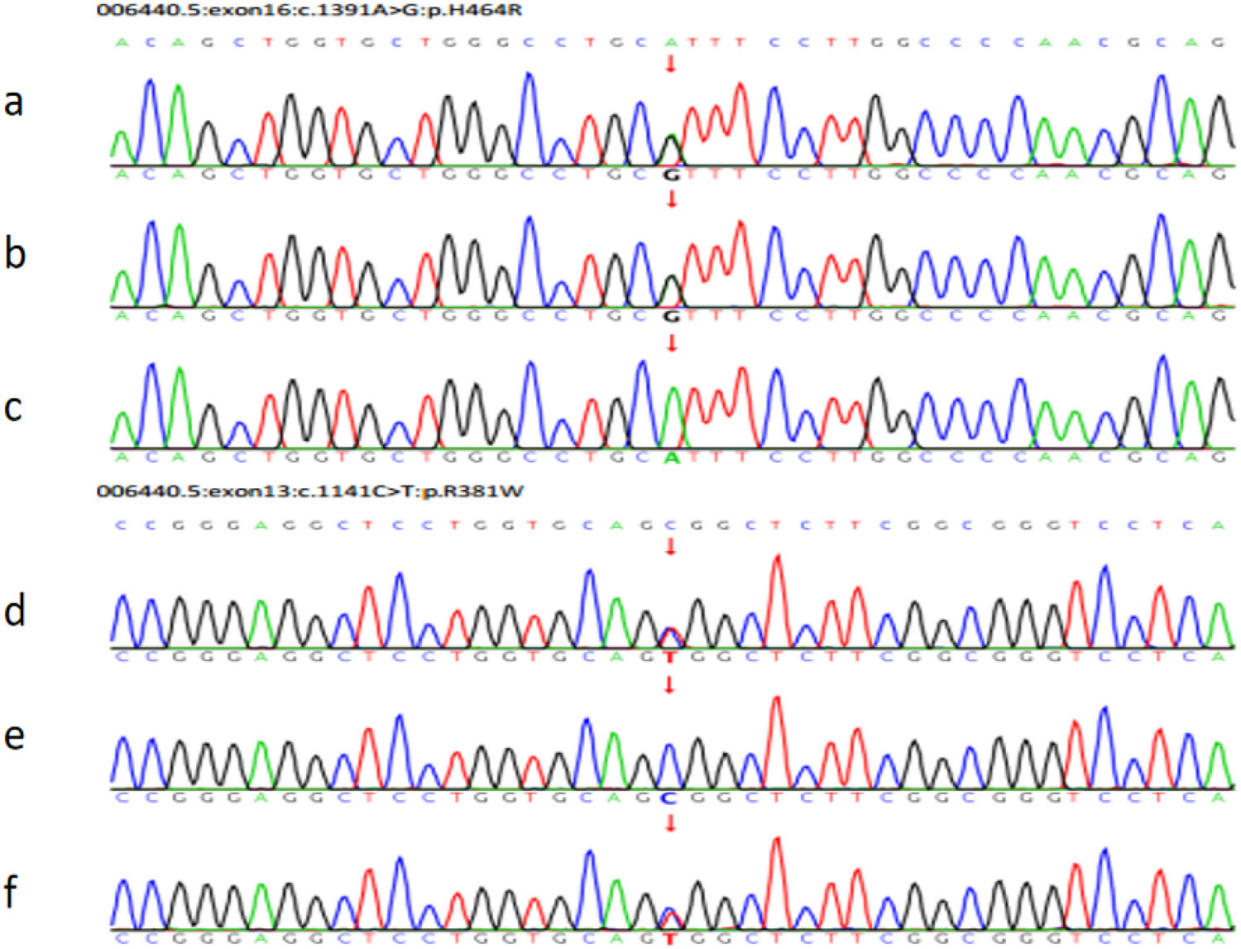
The pedigree of the FGD5 family with *TXNRD2* (c.1391A>G and c.1141C>T). **(a)** The patient's *TXNRD2* c.1391A>G; **(b)** The father's *TXNRD2* c.1391A>G; **(c)** The mother's *TXNRD2* c.1391A (wild type). **(d)** The patient's *TXNRD2* c.1141C>T; **(e)** The father's *TXNRD2* c.1141C (wild type); **(f)** The mother's *TXNRD2* c.1141C>T.

Trio whole-exome sequencing (WES) was performed to screen for potential pathogenic genetic factors. As the *TXNRD2* gene mutation (c.1391A > G; p.H464R and c.1141C > T; p.R381W) had not been previously reported or included in normal controls or disease databases in this context, Sanger sequencing was also performed on the parents, who were found to carry one variant each in the heterozygous state (Figure 5). According to the ACMG guidelines, one mutation (c.1391A > G; p.H464R) is likely pathogenic (LP), and another mutation (c.1141C > T; p.R381W) is Variant of unknown significance (VUS).

### Functional analysis results

**Figure 6:**
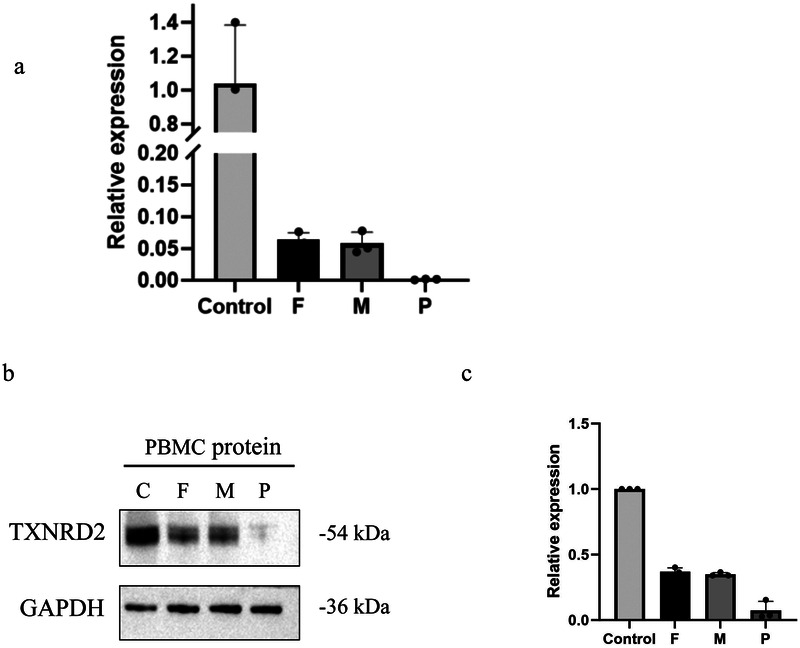
Evaluation the effect of the variant by experiments. **(a)** RT-qPCR demonstrates the effect of mutations on the mRNA expression level of *TXNRD2* genes. **(b,c)** Effects of mutants on TXNRD2 protein levels were detected by Western blot. P: The patient (c.1391A>G p.H464R and c.1141C>T p.R381W), F: the father's TXNRD2 c.1391A>G, M: The mother's TXNRD2 c.1141C>T and control human. Three technical replicates were performed.

Quantitative PCR and western blotting demonstrated significantly reduced TXNRD2 mRNA expression compared to heterozygote carrier parents (Figure 6b), with a corresponding decrease in TXNRD2 protein levels. Further analysis revealed a marked reduction in TXNRD2 gene expression in the patient's (P) peripheral blood relative to both the father (F) and mother (M) (Figure 6b). Consistently, Western blot assays demonstrated substantially lower TXNRD2 protein expression in P's peripheral blood mononuclear cells (PBMCs) compared to those of F and M (Figure 6c).

### Treatments

Based on the results of gene sequencing and clinical features, the patient was considered to have FGD5 caused by the *TXNRD2* gene mutation. Hydrocortisone(HC) acetate is the first choice for children, and the recommended HC dose is 8∼10 mg/(m^2^·d), administered according to the circadian rhythm. The purpose is to ensure normal growth and development and maintain electrolyte balance. The current hydrocortisone dosage for our children is 7.5 mg (8:00), 2.5 mg (16:00), 2.5 mg (22:00), the corticosteroid in the morning rose to 571 nmol/L, and the ACTH decreased to 177.6 ng/L in the morning, and the specific hormone situation is referred to Table 1, and there was no significant improvement in skin pigmentation after 3 months of treatment.

**Table 1:** The hormone of of the FGD5 (TXNRD2 c.1391A > G and c.1141C > T).

Period	Morning cortisol (nmol/L)	Adrenocorticotropic hormone am（<46.37 ng/L）	Serum cortisol pm ( nmol/L)	Adrenocorticotropic hormone pm（<46.37 ng/L）
Pre-treatment	<22	>278	<22	17.2
Post- treatment	571	177.56	224	37.61
Reference	138–690	0–10.21	69–345	0–10.21

ACTH, Adrenocorticotropic hormone;.

### Protein structural modeling

**Figure 7:**
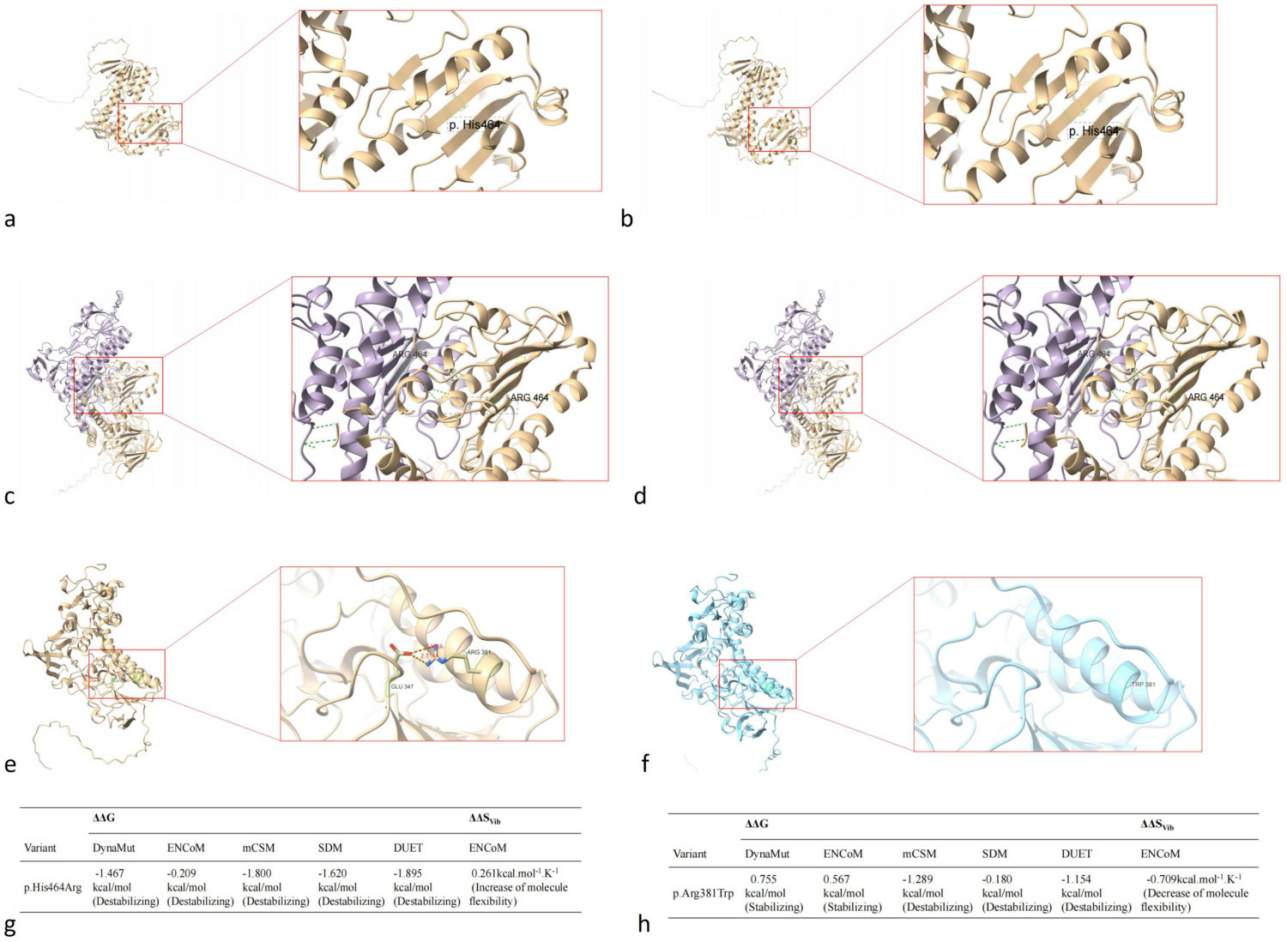
Impact of *TXNRD2* gene mutation (c.1391A > G p.H464R and c.1141C > T p.R381W) on protein structure. **(a)** the tertiary structure of *TXNRD2* p.H464R, **(b)** the tertiary structure of *TXNRD2* p.H464 wild type, **(c)** the quaternary structure of *TXNRD2* p.H464R, **(d)** the quaternary structure of *TXNRD2* p.H464 wild type, **(e)** the tertiary structure of *TXNRD2* p.A381 T, **(f)** the tertiary structure of *TXNRD2* p.A381 wild type, **(g)** the DynaMut website predicted of *TXNRD2* p.H464R, **(h)** the DynaMut website predicted of *TXNRD2* p.A381 T.

Using protein structural modeling analysis, we compared the structural differences between the wild-type *TXNRD2* protein and the two types of mutant *TXNRD2* proteins. The tertiary and quaternary structure of *TXNRD2* p.H464R did not change compared to the wild type, but the DynaMut website predicted that this mutation may lead to reduced protein stability. *TXNRD2* p.Arg381Trp mutation tertiary structure at this location loses 2 hydrogen bonds formed with glutamate at position 347. Predictions on the DynaMut website show that the mutation has an uncertain effect on protein stability (Figure 7).

## Discussion

FGD exhibits genetic heterogeneity, with five main subtypes classified according to their associated genes: FGD1 [melanocortin-2 receptor associated protein (*MC2R*)], FGD2 [melanocortin receptor accessory protein 2 (*MRAP*)], FGD3 [steroidogenic acute regulatory protein (*STAR*)], FGD4 [nicotinamide nucleotide transhydrogenase (*NNT*)], and FGD5 (*TXNRD2*) (4). While *MC2R* and *MRAP* mutations account for approximately 45%–50% of cases, *TXNRD2*-associated FGD5 remains exceptionally rare. Table 2 summarizes characteristics of 4 previously reported FGD5 families with *TXNRD2* variants thus far (5–8) To date, only 10 probands across four independent kindreds with TXNRD2 mutations have been reported globally. Our case represents the fifth documented family and the eleventh proband. Because FGD5 cases are rare, the gene was not reported in the initial report of this patient. Therefore, it is also a reminder to clinicians that the raw data need to be re-analyzed when the genetic report of a patient with glucocorticoid deficiency presents a negative report. *TXNRD2* is one of three thioredoxin reductases, which in mammals are cytosolic (*TXNRD1*), mitochondrial (*TXNRD2*), and testis-specific thioredoxin reductase (*TXNRD3*). Mitochondrial thioredoxin reductase (*TXNRD2*), and mitochondrial thioredoxin and peroxide reducing proteins III and V are essential for the mitochondrial scavenging of reactive oxygen species (ROS), the excess of which can lead to oxidative stress and cell death (9).

**Table 2:** Clinical details of FGD5 cases reported in the previous literature.

ID	Mutation	Sex	Degree of Pigmentation at Presentation	9:00 am Cortisol, nmol/L	9:00 am ACTH	Echocardiogram	Ref
1	c.1341T > G p.Y447X	F	moderate	<10	160 ng/L	Normal	5
2	c.1341T > G p.Y447X	F	serve	<25	500 ng/L	TR and MR	5
3	c.1341T > G p.Y447X	M	none	65	8,130 ng/L	Normal	5
4	c.1341T > G p.Y447X	M	mild	158	514 ng/L	Normal	5
5	c.1341T > G p.Y447X	M	serve	28	3,249 ng/L	Normal	5
6	c.1341T > G p.Y447X	F	none	262	23.2 ng/L	Normal	5
7	c.1341T > G p.Y447X	M	mild	46	>1,240 ng/L	VSD and Truncus arteriosus	5
8	c.1182C > G p.A394l	M	serve	Undetectable	1,070 mU/L	Normal	6
9	c.1321C > T:p.? c.1252C > T:p.R418X	M	serve	Low	NR	Intellectual disability, epilepsy, truncus arteriosus, and omphalocele	7
10	c.1348-1G p.M450Vfs*20	M	NR	<0.3 (21 months)	2,656 pg/ml (21 months)	Bilateral scrotal testes,spastic diplegia,white matter signal abnormality,low vision	8

F, female; M, male; MR, mitral regurgitation; TR, tricuspid regurgitation; VSD, ventricular septal defect: ACTH, Adrenocorticotropic hormone.The 9:00 am cortisol normal range is 138–690 nmol/L, 9:00 am ACTH normal range is less than 10.21 ng/L. Ref, reference; NR: no report.

The thioredoxin (Trx) system consists of Trx, thioredoxin reductase (TR), and reduced nicotinamide adenine dinucleotide phosphate (10). TRX is an important antioxidant molecule that plays a prominent role in redox reactions by resisting the cell death caused by multiple stresses. Trx, thioredoxin reductase (TrxR) and NADPH together form the thioredoxin reductase system, which is involved in many physiological processes (11).

The first step in steroid hormone synthesis is the catalytic conversion of cholesterol-20, 22-desmolase (CYP11A or P450ssc) from cholesterol to pregnenolone (12). This enzyme binds to the inner membrane of mitochondria in all steroid-producing tissues. The adrenal cortisol production pathway is dependent on the energy supply of NADH and NADPH. Thus, the mitochondrial respiratory chain and ROS system are critical to steroid production, and mitochondrial oxidative stress disorder is associated with adrenal gland disease. *TXNRD2* messenger RNA levels are high in the adrenal cortex. Loss of *NNT* gene function leads to decreased NADPH production, resulting in defects in antioxidant defenses, loss of mitochondrial DNA integrity, and reduced mitochondrial mass. *in vitro* models suggests that *TXNRD2* deficiency leads to increased mitochondrial superoxide production, which is not fully compensated for by the glutathione system, resulting in oxidative stress impediment and steroid production. Deficiency in *TXNRD2* impairs the functional integrity of the thioredoxin antioxidant system, leading to the accumulation of reactive oxygen species (ROS). Consequently, this redox imbalance perturbs the glutathione (GSH) metabolic network, as evidenced by a significant shift in the GSH/GSSG equilibrium ratio, reflecting systemic oxidative stress burden. Therefore, *TXNRD2* and *NNT* mutations result in glucocorticoid deficiency, but the exact mechanism underlying this relationship is unknown (13).

*TXNRD2* mutations demonstrate tissue-selective pathogenicity, primarily targeting adrenal fasciculata while sparing other high-energy organs—a stark contrast to systemic mitochondrial disorders caused by *TXNRD2* defects (e.g., cardiomyopathy in encephalopathic forms). *TXNRD2* deficiency disrupts glutathione regeneration, potentiating lipid peroxidation and cytochrome P450scc inactivation (14). Due to cardiomyocytes being highly dependent on mitochondrial energy metabolism, *TXNRD2* may play a key role in the progression of heart failure in patients with ischemic heart disease, hereditary cardiomyopathy, or other diseases. In mice, *TXNRD2* is essential for normal cardiac development and function, with mutations leading to mitochondrial degeneration, which in turn reduces myocardial contractility (15).

Gene mutations in the *TXNRD2* have been linked to rare cases of dilated cardiomyopathy (1.3%) (16), and heterozygous carriers of *TXNRD2* gene mutations have also been reported to have dilated cardiomyopathy, but no individuals with pure or variant *TXNRD2* exhibited any signs of cardiomyopathy or conduction disease (17).

Notably, our patient exhibited electrocardiogram abnormalities including corrected QT interval prolongation and premature atrial contractions. Although previous reports of *TXNRD2*-associated FGD5 did not describe cardiac manifestations, this observation aligns with emerging evidence of *TXNRD2*'s critical role in myocardial redox homeostasis. Cardiomyocytes, being highly dependent on mitochondrial energy metabolism, require efficient reactive oxygen species scavenging systems. Murine models with *TXNRD2* deficiency develop fatal dilated cardiomyopathy through mechanisms involving mitochondrial degeneration and impaired contractility. While human heterozygous carriers typically show normal cardiac function, our findings suggest that biallelic *TXNRD2* mutations might predispose to subclinical conduction abnormalities, warranting longitudinal cardiac monitoring in FGD5 patients. Heterozygous carriers of *TXNRD2* mutations have normal adrenal function, and haplotype insufficiency of *TXNRD2* does not result in adrenal phenotypic abnormalities. The details of our case are consistent with those in previous cases of the *TXNRD2* mutation with only glucocorticoid deficiency and no mineralocorticoid deficiency.

The compound heterozygous variants identified in this case (c.1391A > G; p.H464R and c.1141C > T; p.R381W) occur in critical functional domains. The p.H464R substitution affects a conserved residue in the selenocysteine insertion sequence domain, essential for catalytic activity. The p.R381W variant disrupts a structurally important region adjacent to the flavin adenine dinucleotide binding pocket. Functional studies confirmed these mutations reduce *TXNRD2* protein stability and expression, consistent with previous reports of pathogenic *TXNRD2* variants causing impaired enzyme dimerization and NADPH binding. Our case harboured compound heterozygous variants in *TXNRD2* and abnormal ECG findings, which have not been reported previously, but the related mechanism is unknown.

Diagnostically, this case underscores the importance of re-evaluating negative genetic reports in patients with glucocorticoid deficiency. Initial testing frequently focuses on *MC2R* and *MRAP* mutations, potentially overlooking rare causes like *TXNRD2* defects. mineralocorticoid status, sexual development, and thyroid function remain normal in this case at the time of writing, and long-term follow-up is still required. Long-term management includes: Hydrocortisone replacement (10.8 mg/m^2^/day),biannual cardiac monitoring for conduction abnormalities, annual assessment of adrenal function and electrolytes,the parents reported improved energy levels but expressed concern regarding persistent hyperpigmentation. FGD is a treatable condition that is often missed due to nonspecific findings. If the diagnosis of this disease is delayed, it can lead to adrenal crisis and even death caused by severe infection. Once diagnosed, lifelong follow-up and treatment are required. Therapeutically, our patient required higher-than-standard hydrocortisone dosing (10.8 mg/m^2^/day vs. recommended 8–10 mg/m^2^/day) to normalize adrenocorticotropic hormone levels, suggesting potential interindividual variability in glucocorticoid sensitivity among FGD5 patients. This observation warrants further investigation into genotype-phenotype correlations and optimal dosing strategies for *TXNRD2*-associated adrenal insufficiency.

### Summary

This study expands the *TXNRD2* variant spectrum and highlights the importance of considering FGD5 in patients with isolated glucocorticoid deficiency, particularly those with ECG abnormalities. Reanalysis of negative genetic reports using updated databases is crucial for diagnosis. Long-term monitoring for cardiac complications is recommended.

## Data Availability

The datasets presented in this study can be found in online repositories. The names of the repository/repositories and accession number(s) can be found in the article/Supplementary Material.
