# Supplementary figures and images for: Case report and literature review: novel TXNRD2 compound heterozygous variants in familial glucocorticoid deficiency type 5
Source: Front Pediatr. 2025 Jul 14;13:1585582. doi: 10.3389/fped.2025.1585582 (PMC12301317; doi:10.3389/fped.2025.1585582)

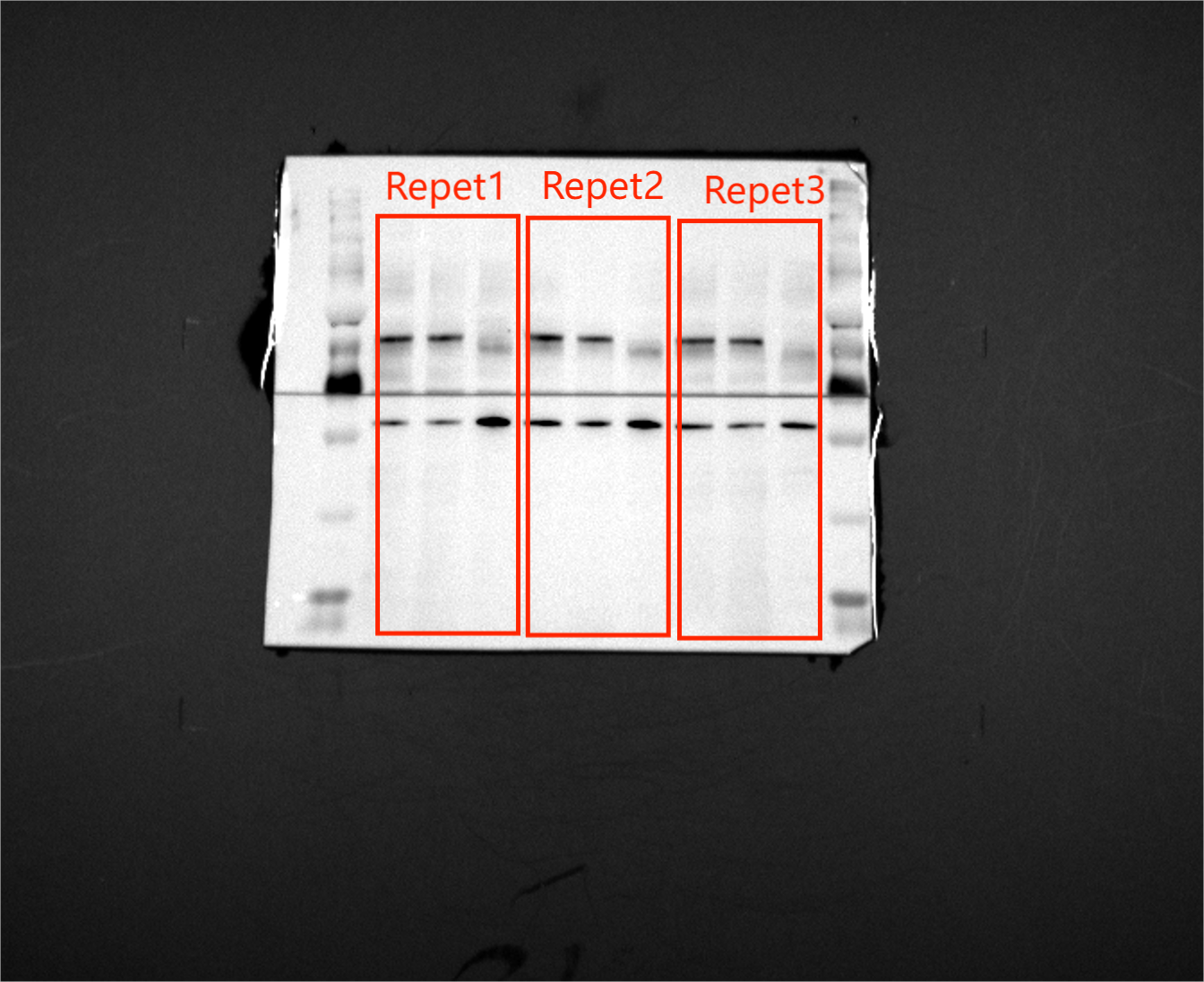

Supplement: Supplementary file 1 [file Image1.png]

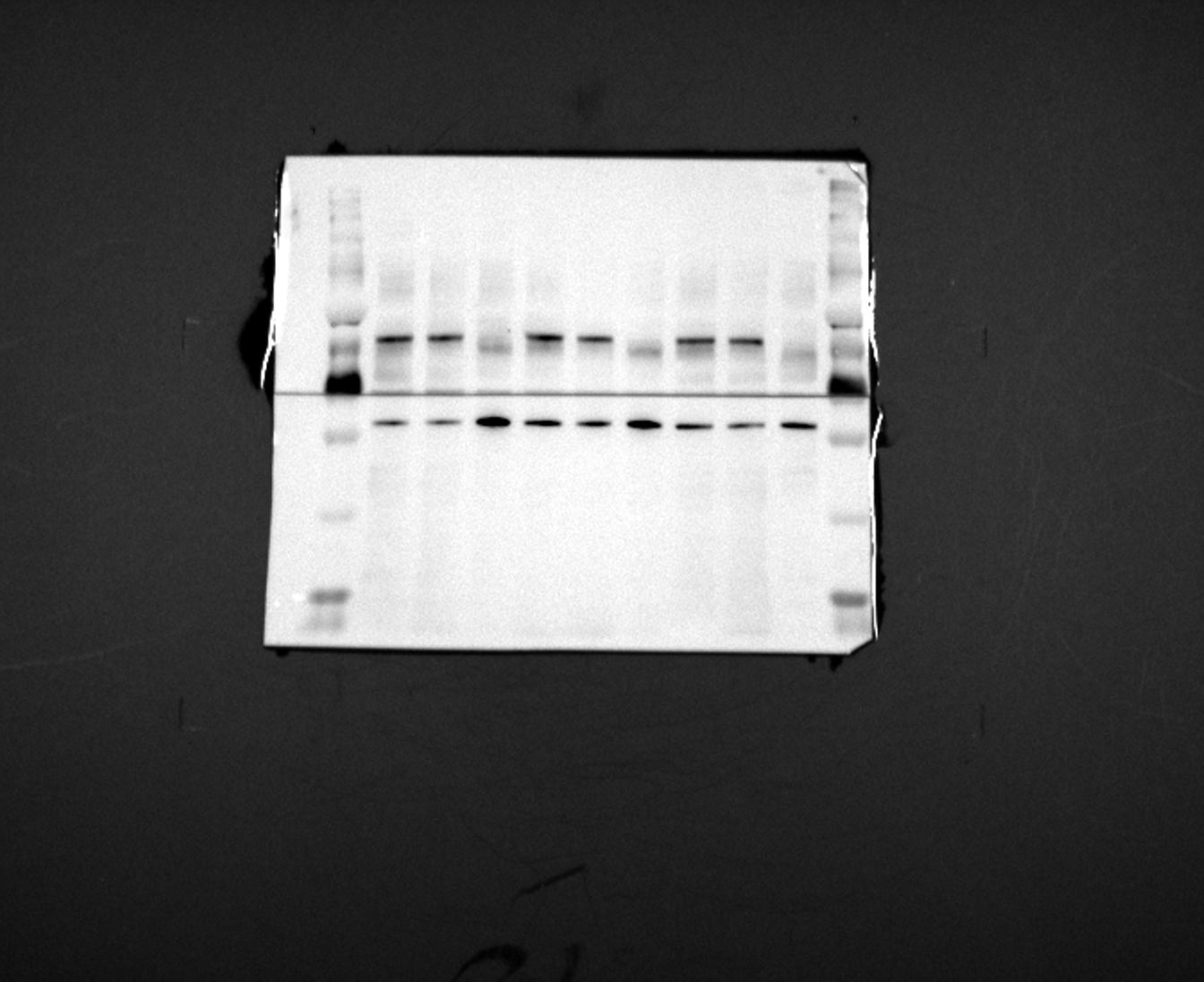

Supplement: Supplementary file 2 [file Image2.tif]
